# Health-related quality of life in epilepsy patients receiving anti-epileptic drugs at National Referral Hospitals in Uganda: a cross-sectional study

**DOI:** 10.1186/1477-7525-12-49

**Published:** 2014-04-12

**Authors:** Anne M Nabukenya, Joseph KB Matovu, Fred Wabwire-Mangen, Rhoda K Wanyenze, Fredrick Makumbi

**Affiliations:** 1 MakSPH-CDC Fellowship Program, Makerere University School of Public Health, P.O. Box 7072, Kampala, Uganda; 2 Department of Epidemiology and Biostatistics, Makerere University School of Public Health, P.O Box 7062, Kampala, Uganda; 3 Department of Disease Control and Environmental Health, Makerere University School of Public Health, P.O Box 7062, Kampala, Uganda

**Keywords:** Epilepsy, Seizures, Anti-epileptic drugs, QOLIE and health related quality of life

## Abstract

**Background:**

Epilepsy is a devastating disorder that impacts on patients’ quality of life, irrespective of use of anti epileptic drugs (AEDs). This study estimates the health-related quality of life (HRQOL) and its associated predictors among epilepsy patients receiving AEDs.

**Methods:**

A total of 175 epilepsy patients already receiving AED for at least 3 months were randomly selected and interviewed from mental clinics at Mulago and Butabika national referral hospitals in Uganda between May - July 2011. A HRQOL index, the primary outcome, was constructed using items from Quality Of Life in Epilepsy Inventory (QOLIE-31) and the Hospital Anxiety and Depression Scale (HADS) questionnaires. The internal consistency and adequacy of these items was also computed using Cronbach's alpha and Kaiser-Meyer-Olkin tests. Partial correlations were used to evaluate the contribution of the health dimensions (mental, psychological, social, physical functioning and emotional well being) and, multiple linear regressions to determine factors independently associated with HRQOL.

**Results:**

Just about half of the respondents (54%) were males, and nearly two thirds (62%) had received AEDs for at least 12 months. The average age was 26.6 years (SD = 11.1). The overall HRQOL mean score was 58 (SD = 13) on a scale of 0–100. The average scores of different dimensions or subscales ranged from 41 (physical) to 65 (psychological). At least three quarters (75%) of all subscales had good internal consistency and adequacy. The largest variations in the overall HRQOL were explained by social and mental functioning; each accounting for about 30% of the difference in the HRQOL but seizure control features explained a little (6%) variation. Factors negatively associated with HRQOL were poly-therapy (-1.16, p = 0.01) and frequency of seizures (-2.29, p = 0.00). Other factors associated with overall HRQOL included drug side effects, sex, marital status and education. Duration on AEDs was not a significant predictor of HRQOL.

**Conclusion:**

The HRQOL for epilepsy patients on AEDs is very low. The predictors of low HRQOL were socio factors (marital status, education) and drug side effects, frequency of seizure, and type of therapy.

## Introduction

The prevalence of epilepsy, a chronic disorder characterized by recurring seizures [1-3], is estimated to be 3% in Uganda [4]. In addition to the unpredictable seizures, some epileptic patients suffer with adverse effects of antiepileptic drugs (AEDs), stigma, psychiatric co-morbidities, social or physical limitations and the possibility of sudden unexpected death [1,5]. Epilepsy is associated with complex effects on social, behavioral, physical, and psychological wellbeing [1].

Whereas anti-epileptic drugs effectively control epilepsy [3,6] in some patients, some AEDs fail while others require poly-therapy (a combination of drugs) to enhance the seizure control [6,7]. The AEDs’ side effects, especially for poly-therapies, have been shown to be negatively associated with health-related quality of life (HRQOL), independent of seizure frequency [8]. Indeed other factors such as stigma [9], depression, anxiety and stress [10] and mood disorders [11] have been found to influence the quality of life of the patients with epilepsy more than seizure severity and frequency control. For this reason HRQOL has been suggested as the most reliable approach for treatment and care of epilepsy. Quality of life (QOL) addresses the different aspects of an individual’s health status. However, in most developing countries such as Uganda, epilepsy treatment has largely focused on reducing seizures severity and frequencies as documented in Uganda National Clinical Guidelines [12].

There are several studies that have evaluated relative contributions of the physical, psychosocial functioning, seizure-related factors and the qualities of life in patients with epilepsy. Some of these studies have obtained contradicting results about the role of these factors on HRQOL. For example, Tracy et al. [13] found that depression, and factors such as seizure control exert a more limited effect on the HRQOL in epilepsy patients. Bautista et al. [14] found a correlation between seizure severity and HRQOL in people with epilepsy independent of seizure frequency, while Vickrey et al. [14] found only minimal correlation between seizure severity and the HRQOL of adult patients. Few studies have been done in Sub-Saharan Africa even though majority of patients with epilepsy (PWE) live there [3]. The HRQOL scores depend on the cultural, ethnic and state of economy and therefore likely to vary from country to country [6] and consequently results from elsewhere are less likely to be generalisable to Ugandan population with epilepsy.

In this study, we estimated the HRQOL and assessed the predictors of HRQOL outcomes in PWE receiving antiepileptic drugs.

## Methods and materials

### Study design and population

A cross-sectional study conducted in two mental clinics at Mulago and Butabika national referral hospitals in Uganda between May to July 2011. Mulago is the national referral hospital and Butabika is the psychiatric national referral hospital. The study population was all people aged 15 years and above who had been on anti- epileptic treatment for at least 3 months. We chose a cut off of 3 months with an expectation that patients would have gotten a good experience on AEDs and possibly would knowledgeably describe seizure control features. A sample size of 177 patients was estimated for both hospitals combined and was calculated to ensure that the overall HRQOL is estimated to within ± 3.25 error of the true HRQOL (on 0–100 scale) with 95% Confidence interval, assuming non-response rate of 10%. Using systematic sampling, with a sampling interval of 3, patients were enrolled and interviewed on a daily basis from Monday to Friday for a period of 4 weeks in each hospital.

### Evaluation of HRQOL

HRQOL was measured using a combination of Quality Of Life in Epilepsy (QOLIE)-31 and Hospital Anxiety and Depression Scale (HADS) validated questionnaires. Both tools were modified to suit the Ugandan situation based on a manual by Guillemin (Guillemin et al., 1993), translated into local languages before use. The QOLIE-31 questionnaire contains 31 items that measure a patient’s physical, social, mental functioning, emotional wellbeing and seizure control features, while the HADS questionnaire includes items that measure anxiety and depression (psychological functioning). Each question is scored on a scale of 0 to 100, with 100 being the highest score. However, possible categories or response sets for scoring vary across questions. Examples of response sets used include (i) 0, 25, 50, 75, 100; ( ii) 0, 20, 40, 60, 80, 100; (iii) 0, 33, 67, 100. Questions on patient socio-demographics and some clinical characteristics (such as partial or generalised epilepsy type) were also included in the integrated questionnaire. Medical records were accessed to extract additional information pertaining to the date of initiation of AEDs and type of therapy (poly-therapy or mono-therapy).

Physical functioning was defined to encompass feelings of fatigue or lack of energy to do activities of daily living including taking care of personal hygiene, travel and execution of job related tasks and explored using a set of 7 questions. Socio-functioning (i.e. social limitations or withdrawal and feelings of stigma) was measured using 6 questions aimed at assessing presence of feelings of internalized stigma and the patient’s ability to associate or relate with others. Emotional well-being was assessed using 6 questions on the extent of worry over epilepsy and seizure reoccurrence while the mental functioning was based on question items that explored a patient’s ability to reason, memorise important aspects or concentrate on key activities. Psychological functioning was assessed using 11 questions (3 items related to mood disturbances and 8 items related to anxiety and depression). In constructing this psychological functioning score, a score for mood disturbance and score for anxiety and depression were constructed separately basing on the HADS questionnaire manual. The two scores were then combined with weights of 0.3 and 0.7 to reflect number of items in each. In all subscales, the higher the scores, the better the quality of life but a score less than 60 is considered poor. For seizure control features, data were captured on the frequency and severity of seizures and side effects of AEDs. The integrated questionnaire was administered by trained nurses with psychiatric training hired from a private clinic but without links to the two hospitals and technical support was given by the Principal Investigator (AMN). In a limited number of cases where a patient’s responses were not clear to some questions, caretakers were asked to clarify further.

### Statistical analysis

Frequencies and percentages were used to summarise the study sample characteristics including side effects of AEDs, perceived impact of epilepsy on social life and activities and feelings of stigma, among others. To determine the HRQOL of (PWE), the mean and median scores of different items on the QOLIE-31 and HADS questionnaire-based questions were used. The computation of HRQOL subscale scores (mental, social, physical, psychological functioning as well as emotional well being) was based on the QOLIE-31 manual for summarizing items using empirical derived weight. The internal consistency and adequacy of such items were computed using Cronbach's alpha [15] and Kaiser-Meyer-Olkin (KMO) test [16].

Univariate linear regressions were done to assess relationships between the overall HRQOL and each of its subscales, seizure control features, sex, and other demographic factors. Only factors that had an F statistic p-value of 0.1 or less were included in the multiple regression models. Multiple linear regressions were used to assess relative importance of the different factors (seizure control features, HRQOL subscales) on HRQOL.

Similar linear regression analysis was done for each of the subscales, mental, social, emotional, psychological, and physical functioning. For statistical efficiency in the final model, a multivariate regression was used. In this model linear regression models for each subscale were jointly fitted. The level of statistical significance was determined at 5% and all analyses were done in STATA.

### Ethical approval

Ethical clearance was sought from the Makerere University School of Public Health (MaKSPH) and High Degrees Research and Ethics Committee (HDREC). Permission was sought from both hospitals to interview as well as access patients and their medical records. Consenting adults of 18 years and above were enrolled into the study and younger patients (15–17 years) provided assent and consent was obtained from an adult caretaker.

## Results

### Sample characteristics

A total of 175/ 177 (98%) patients participated. Two patients declined the interviews. Of the 175 respondents, 54% were male; only 20% were employed and mainly in the informal sector; 62% had been on AEDs for at least 12 months; 50% were aged below 25 years; 51% had attained primary school education level at most; and majority of the patients (77%) were never married. The average age was 26.6 years (SD = 11.1). Majority of the respondents (84%) had generalized epilepsy and about 80% were on poly-therapy. The respondent socio-demographic characteristics are detailed in Table 1.

**Table 1:** Demographics and clinical characteristics

	**Butabika**		**Mulago**		**Overall**	
**Characteristic**	**N (75)**	**% (100)**	**N (100)**	**% (100)**	**N (175)**	**% (100)**
*Age group*						
15 – 19	23	30.7	24	24.0	47	26.9
20 – 24	23	30.7	30	30.0	53	30.3
25-29	8	10.7	21	21.0	29	16.6
30+	21	28.0	25	25.0	46	20.3
*Gender*						
Male	48	64.0	47	47.0	95	54.3
Female	27	36.0	53	53.0	80	45.7
*Marital status*						
Married	8	10.7	14	14.0	22	12.6
Separated	4	5.3	14	14.0	18	10.3
Never married	63	84.0	72	72.0	135	77.1
*Education level*						
None	12	16.0	11	11.0	23	13.1
Primary	32	42.7	35	35.0	67	38.3
Incomplete secondary	22	29.3	42	42.0	64	36.6
Complete secondary	9	12.0	12	12.0	21	12
*Epilepsy type						
Generalized	70	93.3	76	76.8	146	83.9
Partial	5	6.7	23	23.2	28	16.1
*Type of therapy*						
Mono therapy	28	37.3	8	8.0	36	20.6
Poly-therapy	47	62.7	92	92.0	139	79.4
*Referral to hospital*						
H/worker	13	17.3	53	53.5	66	37.9
Self-referral	46	61.3	46	46.5	92	52.9
Other	16	21.3	0	0.0	16	9.2
*Duration on treatment*						
3-12 months	19	25.3	47	47.0	66	37.7
More than 12 months	56	74.7	53	53.0	109	62.3

*For epilepsy type - data was missing for one participant.

Patients sampled from Butabika and Mulago hospitals were different on some characteristics including age, education level attained, duration of AEDs therapy and type of therapy accessed (Table 1). For instance, 55% of patients from Mulago were aged below 25 years as compared to 51% from Butabika; 89% from Mulago ever attended formal education as compared to only 63% from Butabika hospital. Further, 8% and 53% of the patients sampled from Mulago hospital was on monotherapy and been on AEDs from at least 12 months as compared to only 37% and 75% from Butabika hospital, respectively. Further analyses adjusted for hospital site.

### Overall mean HRQOL and its subscales and drug side effects

The overall HRQOL mean score was as low as 58 (SD = 13; 95% CI: 56–60) and a median of 58 (IQR: 47 – 68) (Table 2). The averages of the different HRQOL subscale scores ranged from 41 to 65, with physical functioning and emotional well-being mean scores below 50. All the subscales and overall HRQOL scores had a good internal consistency and sampling adequacy (at least 75%) on the questionnaire items used. Mood disturbance, anxiety and depression are combined to form psychological functioning subscale.

**Table 2:** Overall HRQOL and its subscale/components scores

**General/Dimension**	**Mean**	**SD**	**Min**	**Max**	**Median**	**IQR**	**Internal consistency**	**Sampling adequacy**
Overall HRQOL	58.1	13.1	28.1	85.4	57.7	21.6	0.75	0.77
*HRQOL dimensions*								
Psychological functioning	64.8	17.0	20.0	97.5	65.0	23.4	0.78	0.76
Emotional well-being	48.6	31.2	0.0	100.0	44.3	45.0	0.90	0.87
Physical functioning	40.9	20.5	0.0	100.0	40.0	30.0	0.79	0.72
Mental functioning	59.5	20.1	15.8	91.7	58.3	34.0	0.87	0.84
Social functioning	61.1	25.2	5.0	100.0	58.0	42.0	0.75	0.77
*Psychological items*								
Mood disturbance	62.2	18.9	20.0	100.0	65.0	25.0		
Anxiety and depression	67.4	20.2	13.3	100.0	73.3	20.0		
Medical side effects	66.5	27.3	0.0	100.0	66.7	33.3		
Self-assessed QOL	61.7	20.4	25.0	100.0	75.0	25.0		

With respect to physical functioning, about 36% of the respondents reported to have had trouble with public transport because of their situation. Majority (85%) were able to manage their personal hygiene. Similarly, about 54% are at least somewhat worried of occurrence of a next seizure; with 60% embarrassed (or worried about societal judgment) and about 70% more worried about the seizure associated injuries. Using worries about embarrassment as a measure for `feelings of stigma’, over 60% respondents would be classified as having stigma.

Using seizure control as a proxy for best clinical outcome, it was observed that there was a negative correlation between seizure suppression and mental functioning (Spearman’s r = -0.23; p-value = 0.071) i.e. patients whose seizure control has not improved since initiating AEDs had better mental functioning. Further, over 30% of respondents reported that they had their memory and concentration affected shortly after taking the AEDS in the past 4 weeks which is a known side effect. In general, 83% patients reported that their quality of life had gotten better after initiating AEDs but even so 61% rated their quality of life to be below average.

### HRQOL and relative importance of different factors and subscales

From multiple linear regressions analysis; poly-therapy and increased frequency of seizures are negatively and significantly associated with overall HRQOL (Table 3). Both social functioning and mental functioning independently explained about 30% (partial correlations ≥ 0.33) of the variation in the HRQOL. The extent of the effect of seizure frequency is it plays a minor (6%) role but the worries about the seizure (and possibly stigma) as captured by emotional well-being explained about 17% of the variation in the overall HRQOL.

**Table 3:** **Multiple linear regression and partial correlations (R**^**2**^ **= 0.97) for overall HRQOL**

**Variable**	Coef*	**SE**	**t-value**	**P-value**	**Partial correlations**
AED side effects	-0.24	0.180	-1.34	0.18	-0.02
Therapy (Poly *vs* Mono)	-1.16	0.466	-2.49	0.01	-0.04
Seizure frequency	-2.29	0.505	-4.53	0.00	-0.06
*Psychological functioning*	0.18	0.014	12.91	0.00	0.18
*Emotional well-being*	0.08	0.007	12.11	0.00	0.17
*Physical functioning*	0.07	0.012	5.82	0.00	0.08
*Mental functioning*	0.27	0.012	22.13	0.00	0.31
*Social functioning*	0.22	0.009	23.27	0.00	0.32
*Intercept*	14.93	1.596	9.35	0.00	

*Adjusted for hospital site.

For socio-demographic factors; being female, married, and having some formal education were significantly associated with better HRQOL (Table 4). However, being on poly-therapy, having AED side effects and high seizure frequency were negatively associated with HRQOL. Similarly, patients in Mulago hospital had better HRQOL than those from Butabika hospital. Being unmarried was significantly associated with lower quality of life (t = -2.13; p = 0.03) while age did not predict the overall HRQOL (p-value = 0.41). Duration on AEDs was not significantly associated with HRQOL. Marital status, education level, poly-therapy, AEDs side effects and Site were significantly associated with mental and social functioning. Poly-therapy, AEDs side effects and Site are significant. On the other hand, seizure frequency, hospital and AEDs side effects were significantly associated with physical and emotional wellbeing. For the psychological functioning subscale, only seizure frequency and AEDs side effects were significant. In general the predictors of social and mental functioning were the same as those of the overall HRQOL.

**Table 4:** Multiple linear regressions beta - coefficients for potential factors associated with HRQOL and its subscale scores

**Variable**	**Psychological functioning (R**^**2**^ **= 0.22)**	**Emotional functioning (R**^**2**^ **= 0.32)**	**Physical functioning (R**^**2**^ **= 0.41)**	**Mental functioning (R**^**2**^ **= 0.29)**	**Social functioning (R**^**2**^ **= 0.29)**	**Overall HRQOL ** **(R**^**2**^ **= 0.37)**
Age	0.11	0.33	0.06	-0.09	0.13	0.08
**Sex (Female vs Male)**	3.00	7.65	-3.77	4.77	6.68	4.34*
**Marital status**						
Separated vs married	-3.55	2.51	-0.81	-0.68	-8.27	-3.25
Not married vs married	-1.84	-5.30	-2.00	-10.14*	-10.74*	-5.94*
**Highest education level attained**						
Primary education vs none	3.15	-2.37	-4.59	10.04*	14.96*	6.14*
Incomplete secondary vs none	5.79	-4.44	-5.25	16.02*	25.02*	9.63*
Complete secondary vs none	2.45	-9.38	-4.44	10.24	26.07*	7.97*
**Therapy (Mono vs Poly-therapy)**	1.10	6.82	-5.77	5.44	8.38*	3.12*
**Seizure frequency**	-6.31*	-15.16*	7.48*	7.69*	-3.68	-6.85*
**AEDs side effects**	-3.56*	-4.63*	4.65*	-4.20*	-3.53*	-2.65*
**Hospital site Butabika vs. Mulago**	-2.49	30.93*	13.56*	12.89*	14.19*	8.02*
**Intercept**	74.12	4.75	18.81	41.86	15.45	40.76

*Indicates significance at 5% level with tests carried on variables that have been transformed to normal distribution.

## Discussion

In this study, we estimated the HRQOL score for People with Epilepsy (PWE) in Uganda who had been on AEDs for at least three months and also assessed the relative importance of the different HRQOL sub-scores and associated predictors. This study showed that the HRQOL mean score among the PWEs on AEDs in Uganda is low, at 58 (on 0 – 100 scale). This low HRQOL score implies that these PWEs have poor physical, psychological and mental functioning and poor emotional well-being. Compared to the HRQOL subscales scores reported among TB patients in Uganda in 2010 [17], the PWEs have considerably lower HRQOL scores. Based on a study by Babikako et al. [15], the social, physical and mental functioning average scores for TB patients in Uganda are estimated to be above 70. Similarly, the HIV patients [18] in Uganda have above 80 average score for physical and social functioning and their mental functioning score (average) is above 65. Research in other countries has shown similarly low HRQOL among the PWEs as compared to patients with other chronic conditions [19-22]. In Italy and India, this was attributed to low esteem and stigma and impact of epilepsy on all dimensions of life.

Physical functioning and emotional well-being scores are the most affected HRQOL domains despite PWEs being on AEDs. This is likely because, despite reduced seizure frequency, many physical activities are usually restricted for fear of seizure occurrence. Further, over 60% of the patients could be classified as having stigma (possibly internal stigma) which is expected among PWEs as reported by Jacob [9]. This explains the poor emotional well-being and highlights the fact that HRQOL of PWEs has not improved.

Social, mental and psychological functioning play a major role in contributing to the HRQOL in PWEs and other patients with chronic conditions. The low scores in this domain significantly depress the overall HRQOL. For patients who are on treatment, such as in this study, these domains are more important than seizure feature controls alone (side effects, nature of therapy and seizure frequency). This could be because even if seizure are controlled it cannot necessary improve the social limitations such as chances of marriage. Similarly, people who are stigmatized might not adhere with the AEDs therapy and hence might achieve limited seizure frequency control. Thus, low social functioning and low emotional-wellbeing are likely due to stigma arising from the public, and some family members’ lack of knowledge about epilepsy. This implies that without addressing these other dimensions of HRQOL, the full benefits from treatment with AEDs cannot be realized. In deed studies elsewhere have also shown that treatment with AEDs alone does not significantly improve the social and psychological functioning of PWEs [23].

Mental functioning can actually be depressed for patients on poly-therapy as seen in this study and in other studies elsewhere [7,24,25]. In other words, although clinical management of seizures also aims to reduce on AEDs’ side effects, it is evident that AEDs’ have an effect on mental functioning of some patients and can potentially depress their HRQOL. For example, in our study patients on poly-therapy reported significantly higher number of drug side effects, especially drowsiness, weakness and feeling sleepy immediately after drug taking. This is similar to study reported by Haag et al. [26]. Often poly-therapy is associated with complex dosing regimens which might be bothersome. Secondly, patients on poly-therapy had lower seizure frequency but this was associated with lower mental functioning. Livanainen et al. [27] in their study reported that the continuous administration of some AEDs highly controls seizures but was associated with memory lapse, motor disorientation and aggressive behavior. The implication of this is that a mere focus on seizure control is not entirely beneficial to the overall quality of life of the patient. Management of side effects related to mental functioning will be key to improving the HRQOL of the PWEs on AEDs in Uganda.

Being on AEDs improves HRQOL but the social and mental functioning domains become predominantly important for overall improvement of HRQOL [21]. Low scores for social functioning could be partly attributed to “feelings” of stigma (using embarrassment as a proxy). It has been observed in many studies among PWEs that stigma could lead to psychopathology [28]. Since most the respondents in this study reported embarrassment, it could explain why social functioning played a key role in low HRQOL scores.

The other characteristics of the respondents that impacted on their overall HRQOL positively included gender, marital status and education level. However these factors and seizure control features (seizure frequency reduction, AEDs side effects) only explained 37% variation in the overall HRQOL. This implies that other aspects of overall HRQOL (e.g. social, mental, psychological functioning, etc.) are more important to HRQOL once seizure frequency is moderate. Being young was not substantially associated with low HRQOL. Being married affords one social and psychological support and hence reduces on the negative impact of low scores of these aspects on overall HRQOL. This also highlights the fact that once psychologists and psychiatrists do not address the social, psychological and mental functioning of PWE, family members have to bear these burdens. Social support has been found to be important in many studies [21].

Similarly, education affords one chance to adjust emotions favorably and possibly handle stigma better. Other studies have also reported the positive effect of education [21]. This means helping the children with epilepsy to get education can help to sustain their HRQOL positively.

### Limitations

The study recruited patients who were receiving treatment from the two referral hospitals in Kampala, the capital city. It could be that most of these patients hail from the urban and semi-urban centres where QOL might be better. In addition, because of their conditions, sometimes the PWEs could not understand the questions asked, necessitating use of a caretaker to interpret the questions for them and sometimes clarify on the patients’ responses. There is a possibility that the caretakers did not interpret the questions correctly which might yield a wrong response. However, all the caretakers were oriented on the meaning of the different questions and their comprehension of the questions was assessed. In addition, since the questions were administered in Luganda (the local language) or English – the official language in Uganda – it was possible for the Principal Investigator to correct the caretaker’s interpretation where the actual meaning was being missed by the caretaker.

## Conclusions

The HRQOL mean score of PWEs and receiving AEDs in Uganda is very low (58). It is evident that current management of epilepsy that focuses on only seizure control does not improve HRQOL of the patients receiving AEDs. Treatment of epilepsy should include other health dimensions in addition to seizure control and AEDs side effects. Adding clinical counseling and other interventions to address the physical, mental, psychological, social and emotional aspects for health wellbeing is likely to achieve better health outcomes for epilepsy patients.

## Abbreviations

AED: Anti epileptic drugs; HADS: Healthy anxiety depression scale; HRQOL: Health related quality of life; PWE: People with epilepsy; QOLIE-31: Quality of life in epilepsy – 31.

## Competing interests

The authors declare that they have no competing interests.

## Authors’ contributions

ANM participated in the design of the study, coordination, statistical analysis, and wrote the first draft of the manuscript. JKBM reviewed the manuscript for important intellectual content. FM participated in the writing up of the manuscript and critically analysed the statistical content. FWM supervised the study and also the write up of the first draft. RKW also reviewed the manuscript critically. All authors have read and approved the final manuscript.

## Authors’ information

ANM is currently a MakSPH-CDC Fellow with a Master’s degree in Health Services Research.

JKBM is the Training Manager of the MakSPH-CDC Fellowship Program at Makerere University School of Public Health, Kampala, Uganda.

FM is a senior Lecturer in the Department of Epidemiology and Biostatistics at Makerere University School of Public Health.

FWM is an Associate Professor at Makerere University School of Public Health.

RKW is the Director of the MakSPH –CDC Fellowship Program and Associate Professor in the Department of Disease Control and Environmental Health at Makerere University School of Public Health, Kampala, Uganda.
